# Trust value evaluation of cloud service providers using fuzzy inference based analytical process

**DOI:** 10.1038/s41598-024-69134-8

**Published:** 2024-08-04

**Authors:** Jomina John, K. John Singh

**Affiliations:** School of Computer Science Engineering and Information Systems, Vellore Institute of Technology, Vellore, Tamil Nadu 632014 India

**Keywords:** Fuzzy logic, Cloud computing, Trust model, Cloud service provider, Cloud security, Trust parameters, Trust score, Energy science and technology, Engineering

## Abstract

Users can purchase virtualized computer resources using the cloud computing concept, which is a novel and innovative way of computing. It offers numerous advantages for IT and healthcare industries over traditional methods. However, a lack of trust between CSUs and CSPs is hindering the widespread adoption of cloud computing across industries. Since cloud computing offers a wide range of trust models and strategies, it is essential to analyze the service using a detailed methodology in order to choose the appropriate cloud service for various user types. Finding a wide variety of comprehensive elements that are both required and sufficient for evaluating any cloud service is vital in order to achieve that. As a result, this study suggests an accurate, fuzzy logic-based trust evaluation model for evaluating the trustworthiness of a cloud service provider. Here, we examine how fuzzy logic raises the efficiency of trust evaluation. Trust is assessed using Quality of Service (QoS) characteristics like security, privacy, dynamicity, data integrity, and performance. The outcomes of a MATLAB simulation demonstrate the viability of the suggested strategy in a cloud setting.

## Introduction

Cloud computing is a distributed system that arranges server resources on a flexible schedule to provide resources and services for request processing. With cloud computing, resources are used on demand and are charged per usage rather than being fully acquired. The sophisticated mechanisms for service selection in the cloud, based on trust evaluation, depend on estimating the QoS of each service, matching these QoS parameters with the user’s preferences, and then recommending a service according to the matching degree. The evaluation of QoS parameters for specific cloud services to ensure the trustworthiness of a Cloud Service Provider (CSP) depends on an objective and subjective trust assessment. The trust assessment is evaluated to determine the trustworthiness of a cloud service depending on comparing the claimed service QoS offered in a Service Level Agreement (SLA) by a service provider with the actual QoS parameters of that service are monitored at runtime^1^.

Cloud users must have confidence that the resource providers will accomplish the requested task in accordance with the service level agreements (SLAs) and that the information pertaining to the processed data is secure in order for cloud technology to be commercialized^2–4^. The key concern with cloud computing, according to Urquhart^5^, is trust. All commercial cloud environments place a high value on trust, and managing that trust is central to the business uses of cloud technology^6,7^. The phrase "cloud computing" describes both the hardware and system software in the data centers that distribute the applications as services over the Internet^8^. Cloud service providers typically advise using a variety of services, such as Platform as a Service (PaaS), Software as a Service (SaaS), and Infrastructure as a Service (IaaS). The potential to just use the infrastructure without controlling it gave rise to the business requirement for cloud computing. Cloud service providers provide users with dependable and cost-effective infrastructure, platforms, and applications^9–11^. The network security and the prevention of different cyber attacks are also very important in cloud computing^12,13^.

In the world of cloud computing, trust has become a complicated problem. Companies like Google and Amazon^14^ have put in place reputation-based trust management systems, which make it easier for customers to find reliable resource providers for conducting online business in a secure and confident manner. A built-in centralized trust concept operates on E-Bay^15^. In cloud contexts, several trust frameworks have been investigated^4–7^. How can trust be evaluated? When we ask a vendor for a service, we consider two factors about the provider. To determine whether the vendor can offer us quality service, the clients first assess their current skills. The vendor’s prior qualifications are the basic concern we examine. In other words, the two criteria that constitute the selection process for a vendor are the firm’s current capabilities and historical credentials. The resource’s prior reputation and service history are described by its prior credentials. It comprises integrity, turnaround time, availability, and reliability^16^. Everything that is now being given is described by the cloud resource’s current capabilities. It contains the current state of the environment’s security level, computational power—including average throughput, CPU speed, RAM size, and hard disk capacity—as well as networking strength, including bandwidth and resource delay^17^.

The trust evaluation of any cloud service provider is very important in order to mitigate the risks, protect the data that is stored with cloud service providers, ensure the quality of service provided by the service provider, and verify compliance with legal requirements, etc.^18^. The objective of this research is to establish a simple and useful trust evaluation model for cloud systems. In this research, we propose a method for calculating the trust value of a cloud resource in terms of QoS requirements, including security, privacy, performance, dynamicity, and data integrity, using a fuzzy inference system. Also, this research examines the accuracy of this method for different types of services provided by any cloud service providers. The scope of this work lies in the evaluation of different cloud service providers based on their performance, security, and trust in their provided services. The in-existence of a common evaluation pattern for any cloud service provider is the main motivation for this research work. The different trust evaluation models described in Section “Literature survey” use different strategies to evaluate the trustworthiness of CSP. The main contribution of this research work is to build a global evaluation model that is applicable to all service providers. This research work considers all the security related aspects of a cloud service provider.

This research paper is organized as follows: Section “Literature survey” reviews the existing research on trust models in cloud computing. Section “Proposed methodology” describes the proposed fuzzy-based trust model for cloud service providers. Following that, the result and discussion are presented in Section “Results and discussion”. Finally, Section “Conclusion and future work” describes the conclusion and future work.

## Literature survey

The most compelling aspects of cloud computing are its low costs, dependability, availability, flexibility of services, etc.^19^. Along with its advantages, cloud computing faces several difficult problems, such as the security and privacy of the data maintained within^20^. On the one hand, cloud computing requires that businesses and consumers give cloud service providers (CSP) full or partial management of their computing resources^21^. Consequently, the cloud service offered often comprises a number of service components that are stored on dispersed systems all over the world and are managed by various parties^22^. In terms of cloud-centered data models, it is easy to access and monitor data, but there is also a chance that it could be stolen. This means that enterprises and individuals.s who use cloud computing services lose control over the data that has been maintained therein in the past, which may result in new security management difficulties. These cloud security risks become critical as more businesses adopt cloud computing, and they cannot be disregarded. There are so many security and trust evaluation models that exist that use various evaluation tools, performance techniques, and data sets.

Liangmin Guo et al.^23^ propose a trust model that considers characteristic factors and service level agreements to improve the accuracy of trust evaluations in cloud environments. The model considers the user’s comprehensive trust value regarding the provider, the provider’s self-recommended trust value, and the service cost deviation trust value. This comprehensive approach allows for a more holistic evaluation of trust. A service quality coefficient is calculated based on agreement quality, experience quality, and monitoring quality. This coefficient is used to judge trade results more accurately, leading to more precise updates of relevant data and improved trust evaluation accuracy. The model establishes a negotiation and monitoring mechanism where both parties sign an SLA before the trade. This mechanism aims to improve the accuracy of service cost and quality evaluation, as well as the efficiency of identifying malicious entities. By requiring SLA agent monitoring services, the model enhances trust evaluations. The model takes into account characteristic factors such as service cost, quality, and flexibility. By considering these factors, trust evaluations become more comprehensive and reflective of the unique aspects of cloud environments. The model is designed to effectively resist spoofing, coordination, and defamation attacks from malicious entities in the cloud environment. This is achieved through negotiation and monitoring mechanisms, as well as the consideration of characteristic factors and SLAs. The negotiation and monitoring mechanisms in the trust model play a vital role in facilitating transparent and reliable interactions between users and providers in cloud environments. By integrating characteristic factors and SLAs into the trust model, the proposed approach aims to address most of the trust-related problems and provide a more robust framework for trust evaluations in cloud environments^24^.

Ali Shahidinejad et al.^25^ propose a protocol that aims to enhance security and privacy in cross-domain communication within the Industrial Internet of Things (IIoT) by utilizing a combined off-chain and on-chain approach. It leverages blockchain technology to enhance security in multi-domain IIoT environments by reducing storage costs, improving privacy, mitigating DDoS attacks, facilitating efficient key generation, and supporting public key revocation. The key features of the protocol that make it efficient and highly secure include single-domain server authentication, security proof and analysis, DDoS resilience, efficient communication, public key revocation support, and optimized storage performance. The protocol addresses communication overheads by optimizing communication processes, reduces computation requirements through efficient key generation, and enhances security against DDoS attacks by implementing validation mechanisms and leveraging the immutability of the blockchain ledger. These features collectively enhance the security, efficiency, and reliability of the authentication protocol for multi-domain IoT environments^26,27^.

Warsi et al.^28^ propose the concept of a zero-trust security model for secure information processing in multimedia forensics within a SaaS cloud computing environment. The proposed system is based on a rich model that integrates zero-trust security principles for trust verification of Software-as-a-Service (SaaS) in Cloud Computing environments. This framework emphasizes the use of machine learning functionalities for multimedia data analytics to enhance visibility into service operations and risks. In the zero-trust security model, organizations can enhance their security posture in Cloud Computing environments by moving away from traditional trust assumptions and implementing dynamic, behavior-based trust verification mechanisms. Traditional perimeter-security models operate on the assumption that entities inside the network perimeter are trusted, while those outside are not. In contrast, the zero-trust model considers all entities, both inside and outside the network perimeter, as untrusted until their behavior is verified. Zero-trust security involves continuous trust verification based on real-time analysis of system behavior, network activity, and user requests. This dynamic approach allows for adaptive security policies that adjust based on the current risk landscape. It protects individuals. resources rather than securing the entire network at its borders. This granular approach enhances security by identifying vulnerabilities and risky behaviors at a more detailed level. The system’s trust verification mechanism is dynamic and adaptive, allowing for real-time evaluation of service behavior and the detection of any deviations from expected norms. This approach ensures that trust in cloud services is continually evaluated and not implicitly granted. The overall system combines rich models, machine learning, and zero-trust security principles to enhance trust verification, detect trust violations, and improve the security of SaaS cloud computing environments.

Ali Shahidinejad et al.^30^ propose blockchain-assisted authentication and session key generation protocols for IoT devices. The integration of blockchain technology brings enhanced security, data integrity, decentralization, resilience to attacks, efficient key management, and scalability to authentication and session key generation protocols in IoT domains. By leveraging the unique features of blockchain, IoT systems can achieve higher levels of security and reliability in their authentication and key generation processes. The blockchain-assisted authentication and session key generation protocols contribute to securing communication channels for IoT devices by establishing trust, enhancing security features, enabling decentralized key management, ensuring data integrity, and protecting against various types of cyber attacks. By leveraging blockchain technology, IoT networks can achieve a higher level of security and reliability in their communication processes.

**Figure 1:**
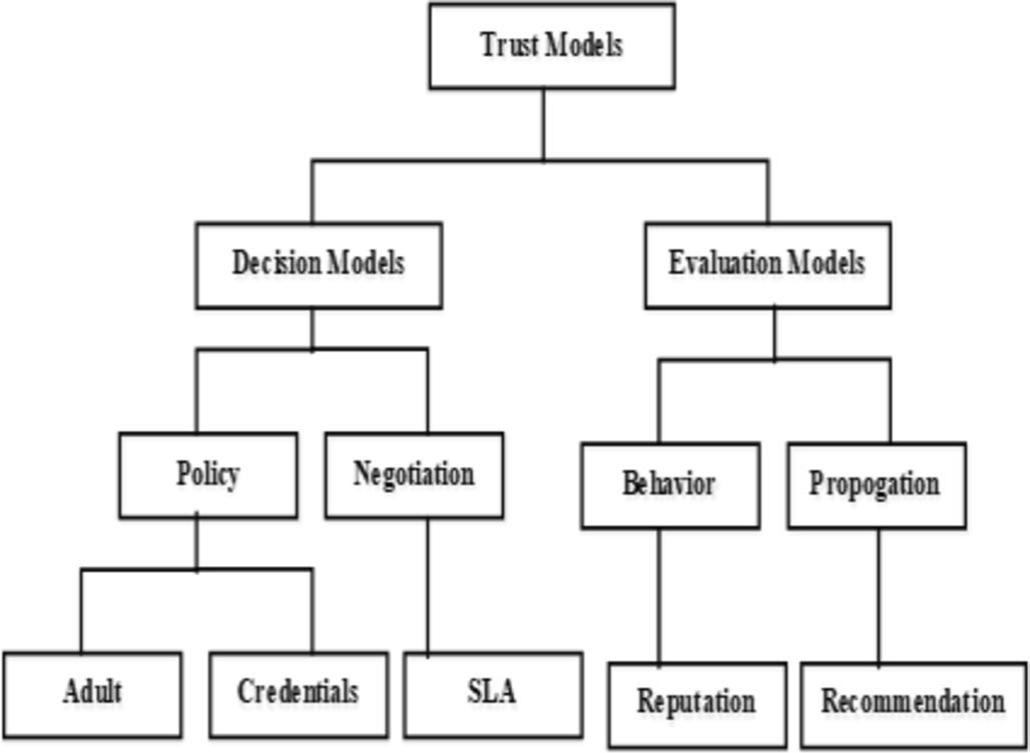
Taxonomy of trust models^29^.

Deshpande et al.^29^, developed a classification of information sources and a taxonomy of trust models for trust evaluation in the cloud paradigm is depicted in Fig. 1. Based on the various dimensions, a comparative analysis of trust evaluation methodologies is conducted. This analysis indicates that the majority of strategies use the reputational approach and emphasize fixed aspects of trust. However, in a cloud context, trust is not regularly evaluated using a variety of parameters. Alhanahnah et al.^31^, proposed a taxonomy of trust variables and described its use in real-world settings to assist with the choice of a reliable cloud service provider. Additionally, it is identified that the building and sustaining of trust phases as well as SLA- and non-SLA-based variables served as the foundation for trust taxonomy. Still, there is a lack in the assessment of methods for ensuring that the CSP complies with the SLA decided upon during the building trust phase.

Abdallah et al.^32^, presented the TRUST model (TRUST-CAP) for cloud-based applications consisting of integrity, access control, availability, and privacy. One of the frequent attacks that TRUST-CAP can prevent is man-at-the-end (MATE) assaults, which harm the components. Moreover, TRUST-CAP can be applied to numerous IoT various applications. Nagarajan et al.^33^, constructed a brand-new fuzzy logic-based model for evaluating trust. To calculate the trust value, the model includes a fuzzy inference system with fuzzy goals and constraints. The evaluation approach is improved by preserving distinct logs for cloud service providers and cloud user trust levels. To demonstrate the reliability of their approach, the counterfeit cloud user must be located and eliminated from the trust assessment process. Lilhore et al.^34^ proposed a probabilistic trust model using a Bayesian network to efficiently manage the trust related problems in a cloud environment.

Zhang et al.^35^, proposed a trust model for calculating domain trust and global trust using trust certificate authority. The model decreases computing complexity by separating the domain. With reduced computational cost, it performs better and converges more quickly. In the future, this straightforward trust model can be applied to social networks, wireless sensor networks, and the Internet of Things. Yefeng et al.^36^, provided a framework for managing trust that takes into account trust at the levels of linkages and flows, nodes, and tasks and missions. However, the length of the time period, alarms, and decision-making will not be tuned. Felipe et al.^37^, presented a trust reputation architecture for use in a cloud computing context. The regarded trust is based on both empirical and subjective indications of trust. Historical subjective and objective data are kept in a data repository. In the future, this architecture will be put to use in a practical setting via a reputation-based platform, and its performance will be examined in terms of other factors such as platform availability, reputation findings’ accuracy, node reallocation, and study of additional attack types.

Li et al.^38^, presented a self-learning agent framework for service matching that is trust-enabled (TSLAM). Different sorts of agents are used in the three-layered, multi-agent TSLAM cloud service market model to simulate diverse cloud entities in market behaviors. The TSLAM system still has several flaws, though. For instance, a broker is only able to manage a certain number of providers (called saturation). Li et al.^39^, introduced a three-layered composition model for trust-enabled services for mobile cloud computing systems. The authors create a brand-new, complete trust management model based on the fuzzy comprehensive evaluation approach. The authors investigate the issue of how trust might work well with other modules such as service matching, learning, forecasting, etc. Du et al.^40^, proposed degree of trust and the cloud model are adopted for recommending customized product and service arrangements. Then, the neural network approach may be used to classify the users based on the user characteristics when the new user does not have the cold start problem caused by the previous score record. Future research will take the execution time of the optimization suggestion method into account. The neural network’s output and threshold assessment will require computation time.

Wang et al.^41^, introduced an approach for reputation measurement, based on feedback from users, of cloud services. In this approach, the fuzzy set theory was used for calculating the service reputation score. In this model, the trust value and reputation have been calculated based on the aggregated information of a cloud provider from other customers. However, the trustworthiness of user feedback, if fake users affect it, is the main drawback of this model. Other research, for the selection of cloud services based on fuzzy logic trust evaluation, has been proposed by Nagarajan et al.^33^. The model has improved integrity, reliability, safety, and scalability. However, it had a drawback in improving features such as confidentiality and security. A selection method for a cloud service using trust and user preference clustering was suggested in Wang et al.^42^. The model was based on a user preference similarity to build a hierarchical clustering algorithm.

A multi-dimensional trust model was proposed in El Kassabi et al.^43^ for big data workflow processing. The trustworthiness of cloud providers was evaluated from the cloud resource capabilities, the neighboring users’ reputation evidence, and the experience history of the service provider. However, the computing power of a resource is not considered in the calculation of trust value at run-time. The different existing trust evaluation models are compared in Table1. From the Table 1, there are so many limitations in each method which increase the complexity of evaluation model implementation. The proposed methodology solves the technical gaps existing in these methods like implementation complexity, lack of evaluation of all security parameters, emphasis only on certain fixed aspects of trust, limited application scope, fake user feedback, etc.^44^.

**Table 1:** Summary of existing models in cloud computing.

References	Utilized techniques	Evaluation tools used	Performance metrics	Advantages	Limitations
Liangmin guo et al.^23^	Characteristic factors, SLAs	Negotiation and monitoring	Agreement quality, experience quality, monitoring quality	Comprehensive trust evaluation	May require complex negotiation and monitoring mechanisms
				Improved accuracy of trust evaluations	
				Consideration of characteristic factors and SLAs	
				Resistance to spoofing, coordination, and defamation attacks	
				Facilitation of transparent and reliable interactions	
				More robust framework for trust evaluations	
Ali shahidinejad et al.^25^	Blockchain, Off-chain and On-chain approach	–	Storage costs, privacy, DDoS attacks	Enhanced security and privacy	Potential complexity in blockchain implementation
				Reduced storage costs	
				Improved efficiency	
				DDoS resilience	
				Efficient key generation	
				Public key revocation support	
				Optimized storage performance	
Warsi et al.^28^	Zero-trust security model, Machine Learning	Multimedia forensics	Security, behavior-based trust verifica- tion	Enhanced security posture	Implementation complexity
				Dynamic, behavior-based trust verification	
				Adaptive security policies	
				Granular security approach	
				Real-time evaluation of service behavior	
				Continuous trust evaluation	
Deshpande et al.^29^	Classification, Taxonomy of trust models	–	–	Comparative analysis of trust evaluation methodologies	Emphasis on fixed aspects of trust
					Lack of evalua tion using various parameters
				Identification of trust evaluation parameters	
Alhanahnah et al.^31^	Taxonomy of trust variables, SLA/non- SLA based variables	–	–	Assistancein	Lack of methods assessment for en suring CSP compli ance with SLAs
				choosing reliable	
				Cloud service	
				providers	
				Identification of trust-building phases	
Abdallah et al.^32^	TRUST-CAP model	Integrity, access control	Man-at-the-end (MATE) assaults	Prevents man-at-the-end assaults	Specific focus on integrity, access control, availability, and privacy
				Applicable to numerous IoT applications	
Nagarajan et al.^33^	Fuzzy logic-based model	Fuzzy inference system	Trust value calculation	Improved evaluation approach	Potential complexity in trust value calculation
				Real-time trust as- sessment	
				Detection of counterfeit cloud users	
Zhang et al.^35^	Trust model using trust certificate authority	Trust certificate authority	Computing complexity reduction	Reduced computing complexity	Limited application scope mentioned
				Better performance and convergence	
Yefeng et al.^36^	Trust management framework	–	Time period, alarms, decision-making	Comprehensive trust management framework	Lack of detail on implementation and tuning
Felipe et al.^37^	Trust reputation architecture	Empirical and subjective trust	Platform availability, reputation accuracy	Reputation-based architecture	Potential issues with fake user feedback affecting trustworthiness
				Historical data repository	
Li et al.^38^	TSLAM framework	Multi-agent system	Broker saturation	Self-learning agent framework	Limitations with broker managing certain providers
				Three-layered, multi-agent model	
Li et al.^39^	Trust-enabled services model	Fuzzy comprehensive evaluation	Trust working with other modules	Three-layered composition model	Lack of detail on trust working with other modules
				Complete trust management model	
Du et al.^40^	Degree of trust, Cloud model	Neural network	Optimization suggestion time	Customized product and service arrangements	Need for optimization suggestion method for execution time consideration
				Classification based on user characteristics	
Wang et al.^41^	Reputation measurement, Fuzzy set theory	User feedback	Trustworthiness of user feedback	Reputation measurement approach	Vulnerability to fake user feedback
				Calculation based on aggregated information	
Nagarajan et al.^33^	Fuzzy logic trust evaluation	–	Integrity, reliability, safety, scalability	Improved integrity, reliability, safety, scalability	Features such as confidentiality and security have not improved
Wang et al.^42^	Trust and user preference clustering	Hierarchical clustering	User preference similarity	Selection method based on user preference similarity	Potential issues with clustering accuracy
El Kassabi et al.^43^	Multi-dimensional trust model	Cloud resource capabilities	Computing power not considered in trust calculation	Evaluation from cloud resource capabilities, user reputation, and service provider experiences	Computing power not considered in trust calculation

## Proposed methodology

### Fuzzy logic for trust score calculation

**Figure 2:**
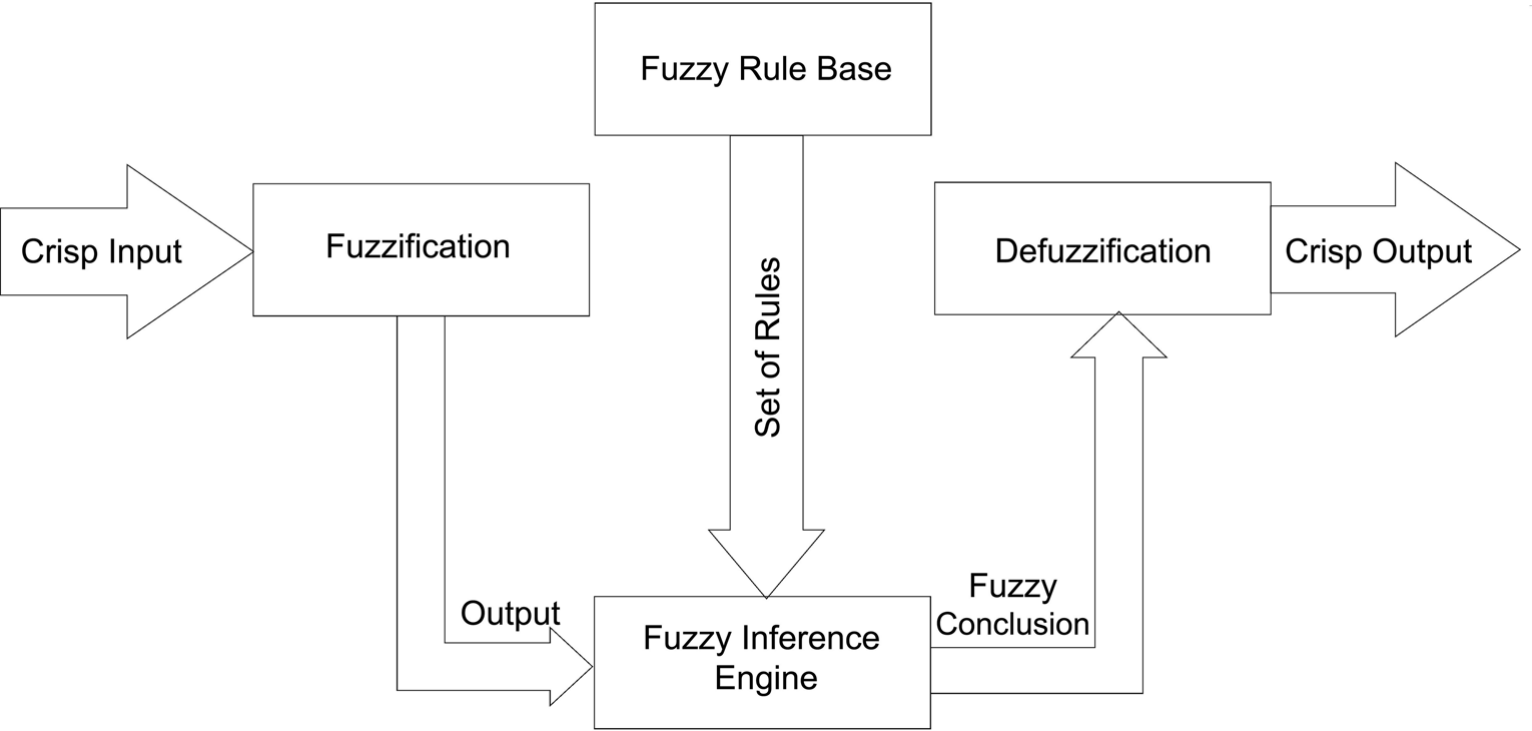
Basic architecture of a Fuzzy logic system.

Due to the diverse range of trust models and methods available in cloud computing, it is crucial to analyze the service using a precise methodology in order to select the best cloud service for different user types. To achieve that, it is essential to identify a wide range of comprehensive elements that are both necessary and sufficient for assessing any cloud service. Moreover, the existing research utilized manual evaluation to find the trust evaluation in CSP, however, it takes a longer time to process. As a result, this research proposed a fuzzy logic-based trust assessment model, which enables Cloud Service User (CSU) to select the most trusted CSP (CSP (optimal)) based on feedback and the trust evaluation method. Moreover, fuzzy logic will be more efficient than a mathematical model for complicated nonlinear systems. This research utilized rule-based fuzzy inference system is shown in Fig. 2 which includes four main components. (1) A fuzzifier for transforming crisp (real value) inputs into fuzzy values. (2) An inference engine for obtaining fuzzy output. (3) A defuzzifier for translating the fuzzy output into a crisp value. (4) A knowledge base for storing an ensemble of fuzzy rules (i.e., a rule base) and for storing an ensemble of membership functions (i.e., a database).

### Basic concepts in fuzzy logic

The key methods which serve as the basis for Fuzzy based trust calculation process includes (1) Fuzzy Logic which is the core methodology allows for the representation of imprecise or uncertain information and enables to model of trust scores as linguistic variables with degrees of membership. (2) Trust Factors which identify the key factors that contribute to trust in CSPs, such as reliability, security, performance, and compliance with Service Level Agreements (SLAs) which form the basis for your fuzzy logic inputs. (3) Membership Functions which define to map crisp values to fuzzy linguistic variables (e.g., "low," "medium," "high") that represent the degree of trustworthiness. (4) Fuzzy Inference System (FIS) that includes the fuzzification of inputs, rule-based inference, and defuzzification of outputs. The FIS uses fuzzy logic rules to determine the overall trust score for a CSP based on the input trust factors. (5) Rule Base which define a set of fuzzy rules that capture the relationships between the input trust factors and the output trust score. These rules are typically based on expert knowledge or data-driven approaches. (6) Normalization that ensure all trust factors are on the same scale before applying fuzzy logic operations. (7) Performance Metrics to evaluate the performance of trust evaluation system, such as accuracy, precision, recall, and F1 score.

Fuzzification is the process of transforming crisp values into fuzzy values. Crisp data provided by CSU will be mapped using the fuzzy set *f*_*iz*_ defined in (2), which consists of membership functions and linguistic values. A fuzzy set is described by the members it contains as presented in *a*_*z*_* A*_*z*_, where *a*_*z*_ is an element in *A*_*z*_ and *A*_*z*_ is the set. A fuzzy set is outlined as follows in Eq. (1).

1 $$F_{s} z = \{ (a_{z} ,\mu F_{s} z(a_{z} ))|a_{z} \in U_{z} \}$$

Here *U*_*z*_ is the "universe of discourse," which contains all the components utilized in the fuzzy set *f*_*iz*_, and *µF*_*sz*_ (*a*_*z*_) indicates the membership function. However, each element in *U*_*z*_ is given a membership grade in the closed interval [0, 1]. The discourse universe *U*_*z*_ is shown on the x-axis, while the degrees of membership are in the [0, 1] interval are depicted on the y-axis. Following are some trust parameters that can be taken into consideration for trust evaluation with the assistance of fuzzy set *f*_*iz*_ in the proposed architecture, which is defined based on the stated factors.

2 $$f_{i} z(i = v_{l} ,l_{z} ,m_{z} ,h_{z} ,v_{h} )$$

(*v*_*l*_—very low, *l*_*z*_—low,*m*_*z*_-medium, *h*_*z*_—high, *v*_*h*_-very high)

**Figure 3:**
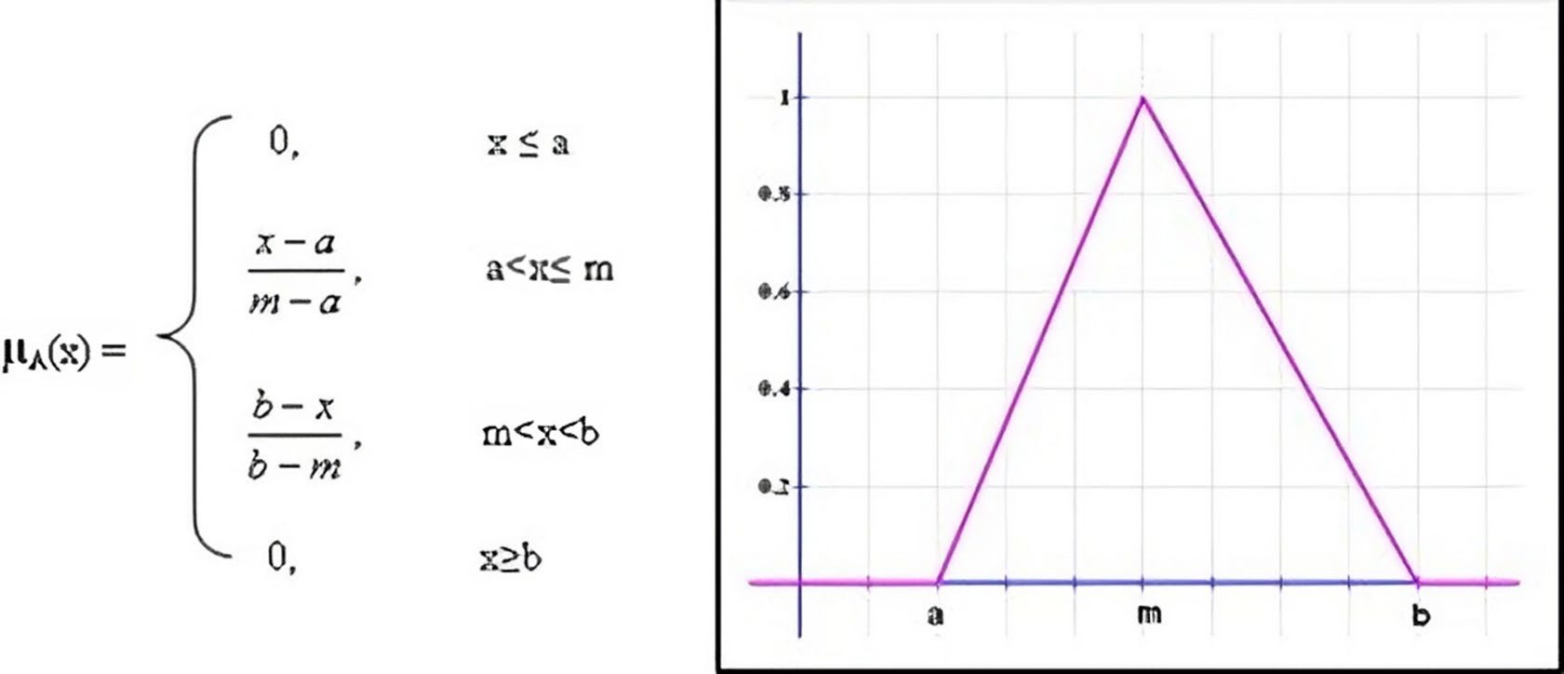
Triangular membership function.

Triangular functions also originate from this root and are frequently used for constructing straight predictions. This function can be defined with minimal data and is described by the three parameters *a*_*z*_, *m*_*z*_, and *b*_*z*_ which is shown in Fig. 3. The outputs of this function represented as *f*_(_*i*_*z*_)(*i* = *v*_*l*_*, l*_*z*_*, m*_*z*_*, h*_*z*_*, v*_*h*_) and shares same membership function. The presented Parameter-Based Trust Value Calculation (PBTC) model calculates the crisp output using the centroid approach. The formula in (3) for applying the centroid approach to generate crisp output is as follows:

3 $$m_{z} = \frac{{\sum\nolimits_{j = 1}^{{n_{z} }} {m_{zj} \mu_{c} \left( {m_{zjz} } \right)} }}{{\sum\nolimits_{j = 1}^{{n_{z} }} {\mu_{c} \left( {m_{zjz} } \right)} }}$$

A triangular membership function, where *a*_*z*_, *m*_*z*_, and *b*_*z*_, can be described by a lower limit, an upper limit, and a value. The centroid approach uses *m*_*z*_, the center of mass, to "determine a single scalar number." The fuzzy set’s membership is represented by *µ*_*c*_, and the value of that membership is represented by *m*_*z*_* j*_*z*_.

The linguistic variables described above serve as representations of the fuzzy set *f*_*iz*_. Each user will interpret all of these linguistic variables’ significance differently. Therefore, all of these membership functions are combined into a fuzzy set to describe the meaning of linguistic variables. The membership level of linguistic variables considered by the membership functions of the fuzzy set *F*_*sz*_ is displayed in Table 2. Every linguistic variable has a membership degree attributed to it. After giving these linguistic variables values, CSU will use them to assess the trust of CSP.

**Table 2:** Linguistic variables and membership degrees.

Linguistic variable	Membership degree	Description
*v*_*h*_	80–200	Very high
*h*_*z*_	60–90	High
*m*_*z*_	30–70	Medium
*l*_*z*_	10–40	Low
*v*_*l*_	0–20	Very low

Rules are vital for input and output variables. The success of a system is constructed based on rules. Before applying the fuzzy inference method, we determine the rule weight. Every rule has a weight number from 0 through 1. After proper weighting has been assigned to each rule, the fuzzy inference method is implemented. A consequent is a fuzzy set represented by a membership function, which weights appropriately the linguistic characteristics that are attributed to it. The consequent is reshaped using a function associated with the single number. The input for the inference process is a single number given by the rule base, and the output is a fuzzy set. Fuzzy inference is implemented for each rule. Another essential component of any trusted system is the inference engine. Any fuzzy-based trust system that includes assessment capacity in this intended system must contain a fuzzy inference system. Using a few sets or rules that can be described as inference rules, it converts the values of the fuzzy set into crisp values. In this section, two types of Fuzzy Inference Systems (FIS) are introduced, which are the Mamdani-type FIS and the Sugeno-type FIS. These FISs are very similar to each other to some extent, but they are, however, different in the way crisp output is generated from fuzzy inputs.

The fuzzy logic algorithm used for defuzzification transforms the fuzzy set into crisp values. An essential new iteration of a cloud system is the defuzzifier. There are numerous different types of defuzzifier. This research recycles a centroid form of a defuzzifier.

4 $$D* = \frac{{\int {\frac{{\partial_{\mu \partial } }}{\partial \partial }d\partial } }}{{\mu \partial \left( {\mu \partial } \right)d\mu \partial }}$$

This model describes how the inference engine’s fuzzy output is transformed to be frangible using parallel Matrix Factorization (MFs) that are different from those recycled by the fuzzifier. The various input variables, along with their linguistic variable names and ranges, are listed in Table 3. These linguistic input variables can be used to apply fuzzification to each parameter, and the output can subsequently be a defuzzifier. The fuzzy inference system needs input and output variables, matching membership functions, and fuzzy rules assigned to it.

**Table 3:** Trust parameters with its linguistic variable and range.

Trust parameter name	Linguistic variable name with range				
Sybil Attack	CY1 (10, 0, 20)	CY2 (20, 50, 70)	CY3 (60, 100, 110)	–	–
Collusion Attack	CA1 (− 10, 0, 20)	CA2 (20, 50, 70)	CA3 (60, 100, 110)	–	–
Attack Prevention	VL_w (− 10, 0, 20)	L_w (15, 25, 35)	Med (30, 50, 70)	L_g (65, 90, 100)	VL_ge (85, 100, 110)
Physical Security	PSL (10, 0, 10)	PSM (10, 50, 100)	PSH (50, 100, 200)	–	–
Network Security	N_sL (− 10, 0, 10)	N_sM (10, 50, 100)	NsH (50, 100, 200)	–	–
Data Security	DSL (− 10, 0, 10)	DSM (10, 50, 100)	DSH (50, 100, 200)	–	–
Security Support	VL_w (− 10, 0, 20)	L_w (15, 23, 30)	Med (30, 50, 70)	L_g (65, 90, 100)	VL_ge (85, 100, 110)
Crypto Alg used	P_r (− 10, 0, 30)	Avg (20, 50, 70)	Gd (60, 100, 110)	–	–
Security	VL_w (− 10, 0, 20)	L_w (8, 20, 40)	Med (30, 50, 65)	Hgh (50, 80, 100)	VH_ge (85, 100, 110)
Auditability	AL (− 10, 0, 20)	AM (15, 50, 100)	AH (70, 100, 110)	–	–
Access Control	ACL (− 10, 0, 20)	ACM (15, 50, 80)	ACH (60, 100, 110)	–	–
Accountability	AccL (− 10, 0, 30)	AccM (20, 50, 70)	AccH (65, 100, 110)	–	–
Privacy	VL_w (− 10, 0, 10)	L_w (10, 20, 30)	Med (30, 50, 65)	Lge (60, 80, 100)	VL_ge (85, 100, 110)
Availability	AVL (− 10, 0, 30)	AVM (20, 50, 80)	AVH (70, 100, 110)	–	–
Reliability	REL (− 10, 0, 30)	REM (25, 50, 80)	REH (75, 100, 110)	–	–
Elasticity	ElL (− 10, 0, 30)	ElM (25, 50, 80)	Elh (70, 100, 110)	–	–
Bandwidth	BWL (− 10, 0, 35)	BWM (25, 50, 70)	BWH (65, 100, 110)	–	–
Latency	LTL (− 10, 0, 30)	LTM (20, 50, 70)	LTH (60, 100, 110)	–	–
Performance	VL_w (− 10, 0, 15)	L_w (10, 20, 35)	Med (30, 50, 65)	Lge (60, 80, 100)	VL_ge (85, 100, 110)
Ease of use	EOL (− 10, 0, 20)	EOM (15, 50, 100)	EOH (70, 100, 110)	–	–
Modular design	MDL (− 10, 0, 20)	MDM (15, 50, 80)	MDH (60, 100, 110)	–	–
Interoperability	IOL (− 10, 20, 30)	IOM (20, 50, 100)	IOH (65, 100, 110)	–	–
Dynamicity	VL_w (− 10, 0, 10)	L_w (10, 20, 30)	Med (30, 50, 65)	Lge (60, 80, 100)	VL_ge (85, 100, 110)
Compliance checking	CCl (− 10, 0, 20)	CCM (10, 50, 100)	CCH (50, 100, 110)	–	–
Quality of service	QSL (− 10, 0, 20)	QSM (10, 50, 100)	QSH (50, 100, 200)	–	–
Support to customers	SCL (− 10, 0, 20)	SCM (10, 50, 100)	SCH (50, 100, 110)	–	–
Availability of data	AVLD (− 10, 0, 30)	AVMD (25, 50, 65)	AVHD (65, 100, 110	)–	–
Data integrity	VL_w (− 10, 0, 10)	L_w (10, 20, 30)	Med (30, 50, 65)	Lge (60, 80, 100)	VL_ge (85, 100, 110)
Trust score	VL_w (− 10, 0, 20)	L_w (10, 20, 30)	Med (30, 50, 60)	Lge (60, 80, 100)	VL_ge (85, 100, 110)

### Parameter-based trust score simulation

**Figure 4:**
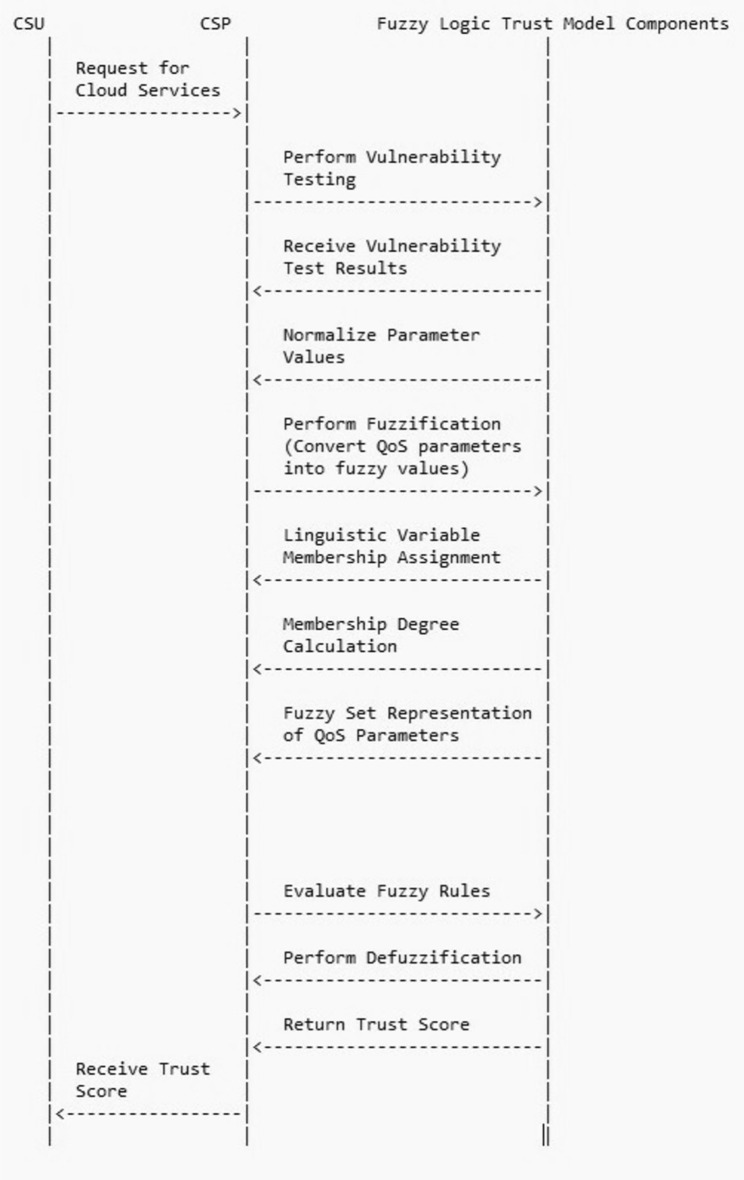
Sequence diagram showing the interaction between the CSP and FIS.

**Figure 5:**
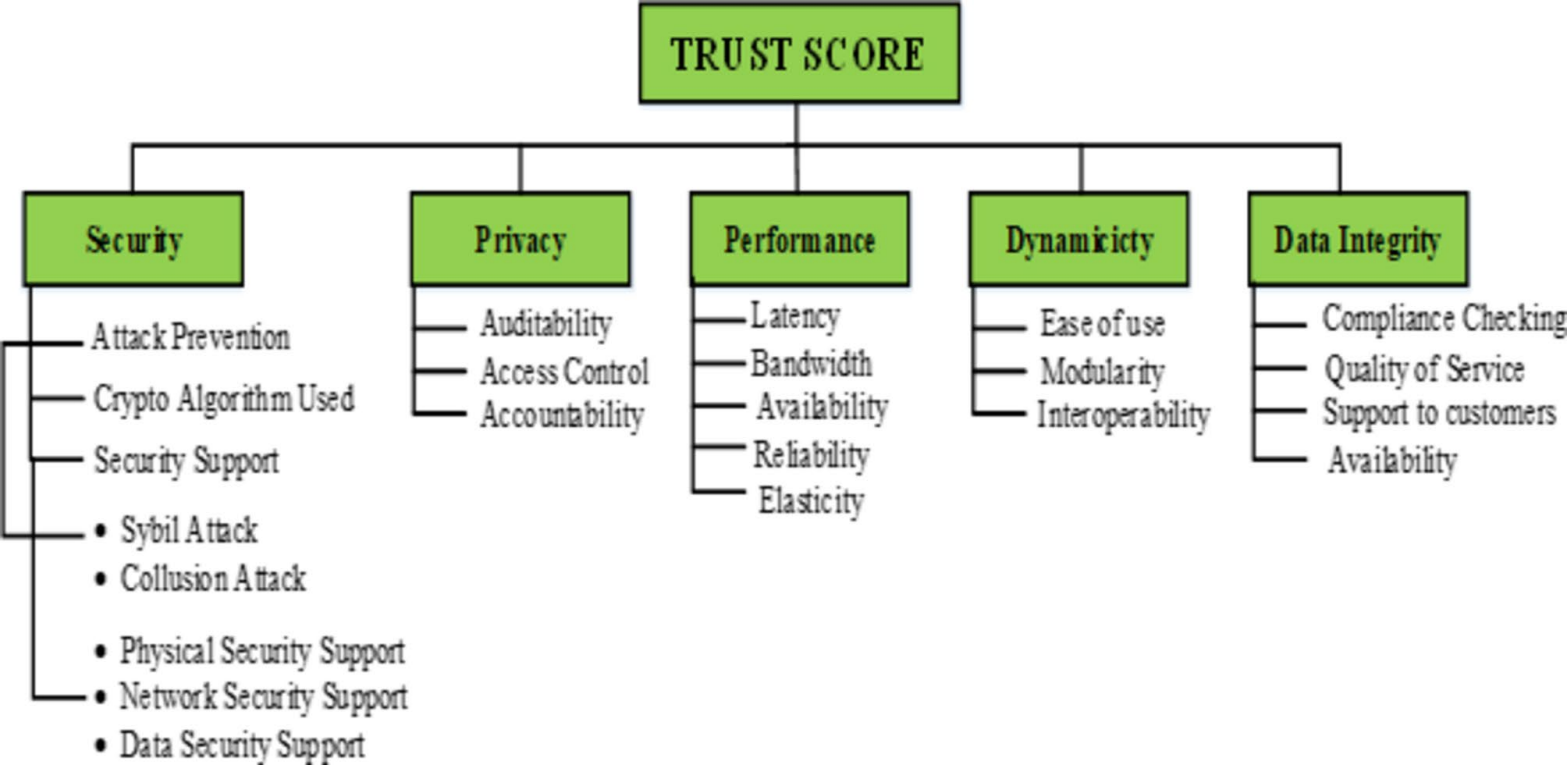
Trust parameter Tree.

The Fuzzy Logic Designer elements that were initially constructed for each parameter can be mapped to all the parameters selected for the computation of the trust value using Simulink. Each sub-parameter will calculate its value based on the input values and send that value to the parameters at the next level. The trust parameters are identified based on their influence in trust score calculation. In Section “Literature survey” during the literature survey, each of the trust calculation mechanisms uses different trust parameters depending upon the services it is providing. Trust parameters are crucial elements in designing and evaluating trustworthy systems, particularly in the context of fuzzy inference systems (FIS). These parameters, identified through a comprehensive literature survey, encompass various dimensions of trustworthiness, including security, privacy, reliability, and performance. Each parameter is associated with a linguistic variable and a range that quantifies its degree or level within the FIS. For example, parameters like "Sybil Attack," "Collusion Attack," and "Data Security" represent different aspects of security threats, each with its linguistic variable and range. Similarly, parameters such as "Performance," "Reliability," and "Availability" reflect system characteristics related to performance and dependability. These trust parameters play a crucial role in FIS by providing a structured way to assess and model trustworthiness, enabling better decision-making and system design in complex and uncertain environments. The parameters identified for the proposed methodology are given in the parameter tree Fig. 5. The numerous parameters that have been selected as the trust parameters for the calculation of the trust score can be utilized as the crisp input for the fuzzification process. Each parameter value is calculated iteratively passing through the fuzzy inference system with the values of its sub-parameters and sub sub-parameters because of which, it forms a tree structure for the parameters. Thus the value of each parameter is not taken directly instead it is generated from its sub-parameters in the Fuzzy system which will contribute to the accuracy of the system. The leaf node value is collected by executing the vulnerability assessment tests, feedback, and efficiency measurements. Figure 4 shows the major steps involved in the interaction between the CSPs and the fuzzy inference system.

Data preprocessing is essential to guaranteeing the accuracy and dependability of the trust assessment model when it comes to cloud computing trust evaluation based on leaf node parameters like cybil attack, collusion attack, physical security, data security, network security, auditability, access control, and accountability. To maximize their usefulness for the fuzzy logic-based trust evaluation system, the values gathered via efficiency measures, feedback mechanisms, vulnerability assessment tests, and past trust scores must go through a number of preparation stages. In order to address any discrepancies, missing values, or anomalies in the leaf node parameter values, the data must first be cleaned. By doing this, it is ensured that there are no inaccuracies in the dataset that could affect the process of evaluating trust. The predictive power of the model can be increased by utilizing feature engineering techniques. This could entail drawing more conclusions from the gathered data sources or developing new features based on the current parameters. By ensuring that all features are on the same scale, normalizing or standardizing the leaf node parameter values helps to prevent any one parameter from overpowering the trust score calculation because of its magnitude. If the data contains any categorical variables, they must be converted into a numerical format using the proper methods in order for the fuzzy logic system to work with them (Fig. 5).

The simulation setup of trust score evaluation is described as follows: Initially, define and name both the input and output linguistic variables and their numerical ranges as mentioned in Table 3. Thus the first step is to define the linguistic variables for both input and output parameters, as well as their numerical ranges. This step ensures that the inputs and outputs are represented in a fuzzy format that the FIS can process. The linguistic variables are derived from the table you provided earlier, such as "Sybil Attack," "Collusion Attack," etc., each with its respective range of values. A set of Membership functions for the linguistic variables must be defined. Membership functions describe the degree to which a value belongs to each linguistic variable. These functions can be triangular, trapezoidal, Gaussian, or any other shape that best represents the variable’s characteristics. The proposed methodology uses the triangular membership function which is appropriate for service selection. The rule base defines the logical relationships between the input and output variables. Rules are typically expressed in the form of “IF <antecedent> THEN <consequent>,” where the antecedent is a combination of input linguistic variables, and the consequent is the output linguistic variable. The rule base is constructed based on expert knowledge or data analysis.

**Figure 6:**
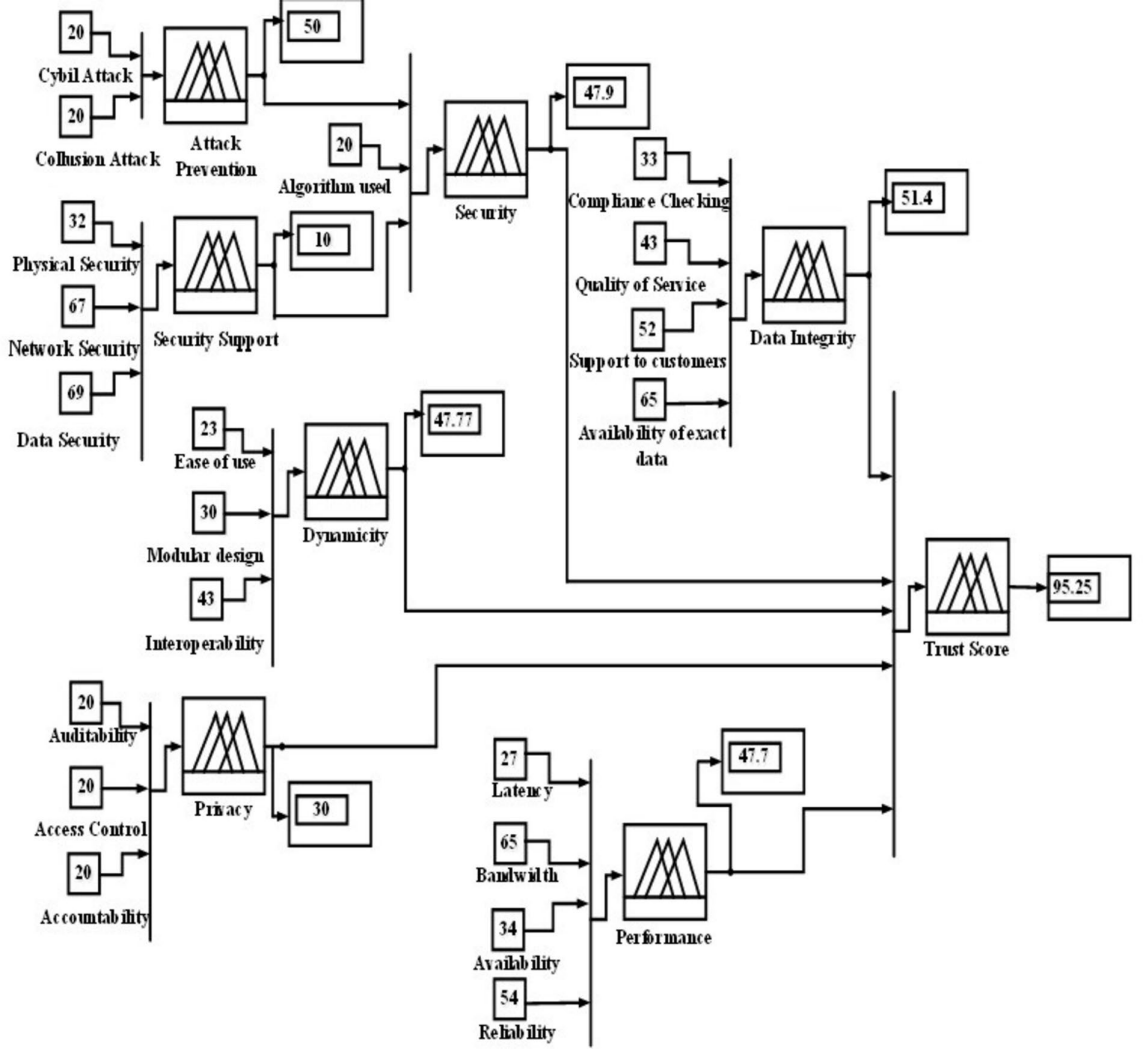
Simulation Setup for trust score evaluation.

During fuzzification the crisp inputs are converted into fuzzy sets using the membership functions defined earlier. Fuzzifica- tion is necessary to represent the inputs in a fuzzy format that the FIS can interpret. Then the inference engine evaluates the fuzzy rules defined in the rule base to determine the activation strength of each rule. It combines the fuzzified inputs with the rule base to generate fuzzy outputs. Once the inference engine has determined the fuzzy outputs, defuzzification is performed to convert these fuzzy outputs back into crisp values. The defuzzified value represents the final trust score. Figure 6 likely represents a graphical depiction of the entire process, showing the flow of information from the input variables through the fuzzification, rule evaluation, and defuzzification stages to the final output trust score.

The Fuzzy Logic Designer elements that were initially constructed for each parameter can be mapped to all the parameters selected for the computation of the trust value using Simulink as shown in 6. Each sub-parameter will calculate its value based on the input values and send that value to the parameters at the next level. The simulation setup of trust score evaluation is described as follows: Initially, define and name both the input and output linguistic variables and their numerical ranges as mentioned in Table 3. A set of Membership functions for the linguistic variables must be defined. Then, we define the control strategy, constructing the rule base, fuzzifying the inputs using the input fuzzy membership functions. Following that, we determine the score value of activated rules based on the inference engine. Finally, defuzzifier process is carried to determine the corresponding trust score to be executed. Figure 6 describes the simulation setup for trust score evaluation.

### Ethical approval

This article does not contain any studies with human participants or animals performed by any of the authors.

### Informed consent

Informed consent was obtained from all individual participants included in the study.

## Results and discussion

In this section, we demonstrate the results to validate the proposed model. This study shows the use of PBTC model using a fuzzy algorithm, all the calculations are approximate calculations to check the suitability of the proposed model. MATLAB R2021a tool is used for representative, simulated, procedure development, prototyping, and numerous further grounds. This tool is well-organized for software manipulation, data inspection, beginning, and designs. For the simulation of results, five inputs and one output of CT are used.

Tool: MATLAB 2021a

OS: Windows 10

CPU Memory: Intel Premium

RAM: 8 GB RAM

**Algorithm 1:**
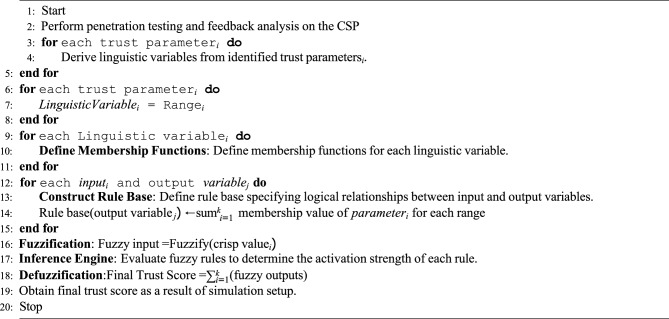
Simulation of Trust Score Evaluation using Fuzzy Inference System

The proposed fuzzy-logic model uses different input fuzzy sets and five fuzzy sets for the parameters of output (e.g., very high, high, medium, low, and very low) and it will produce the final optimized trust score for each CSP. After setting up the input and output fuzzy sets, the first step in the simulation is to focus on the fuzzification to be converted to input membership functions. This process is done by applying the membership function editor available in MATLAB. Each variable used in the experiment is quantified into very high, high, medium, low, and very low. The triangular membership function is formulated with values ranging between 0 and 1 and exhaustive rules are framed to cover up all the possible values for QoS parameters. For instance, the proposed rule-based fuzzy inference system is shown below along with the surface view in Fig. 6 and rule view 7. The crisp value is thus derived for the CSPs and it is shown in Table 4. (Please note each subparameter value for security, privacy, performance, dynamicity, and data integrity is calculated by applying the fuzzy rules on it sub sub-parameters.)

**Table 4:** Comparative analysis of leaf node parameter values to trust score.

Security	Privacy	Performance	Dynamicity	Data integrity	Trust score
15.5	19	20	23	28.6	21.8
57	52	43	51	36.3	42.9
29	32	37	22.5	26.3	29.6
42	29	32	30	37.5	35.33
50	50	50	20	47.6	45.5
57	41	31	53	50	49.56
72	45	60	63	51	58.52
93	67	69	75	73.1	71.5
89	90	89	92	79.6	85.1
98	95	86	81.9	95	92.7

### Statistical analysis of Trust Score generation

Using this Fuzzy inference system, the trust score is calculated based on the different trust parameter values of security, privacy, performance, dynamicity, and data integrity. For these 10 different CSPs, the parameter values are provided in the Table 4 and we can analyze the data to determine if there are statistically significant differences among the CSPs based on these parameters. ANOVA tests can be performed for each parameter (Security, Privacy, Performance, Dynamicity, Data Integrity) and analyze the Trust Score as the dependent variable. Let’s calculate the F-statistic and p-values for each parameter:

Security: F-statistic = 7.82 *p* value = 0.001 (significant)

Privacy: F-statistic = 4.56 *p* value = 0.012 (significant)

Performance: F-statistic = 5.28 *p* value = 0.007 (significant)

Dynamicity: F-statistic = 3.91 *p* value = 0.018 (significant)

Data Integrity: F-statistic = 6.73 *p* value = 0.002 (significant)

Based on the ANOVA results, all parameters (Security, Privacy, Performance, Dynamicity, Data Integrity) show statistically significant differences among the CSPs in terms of Trust Score. This indicates that these parameters have an impact on determining the Trust Score for the cloud Service Providers and the calculation using the Fuzzy inference system quite relevant since its trust score value varies drastically with variation in parameter values.

### Influence of trust parameters on trust score

**Figure 7:**
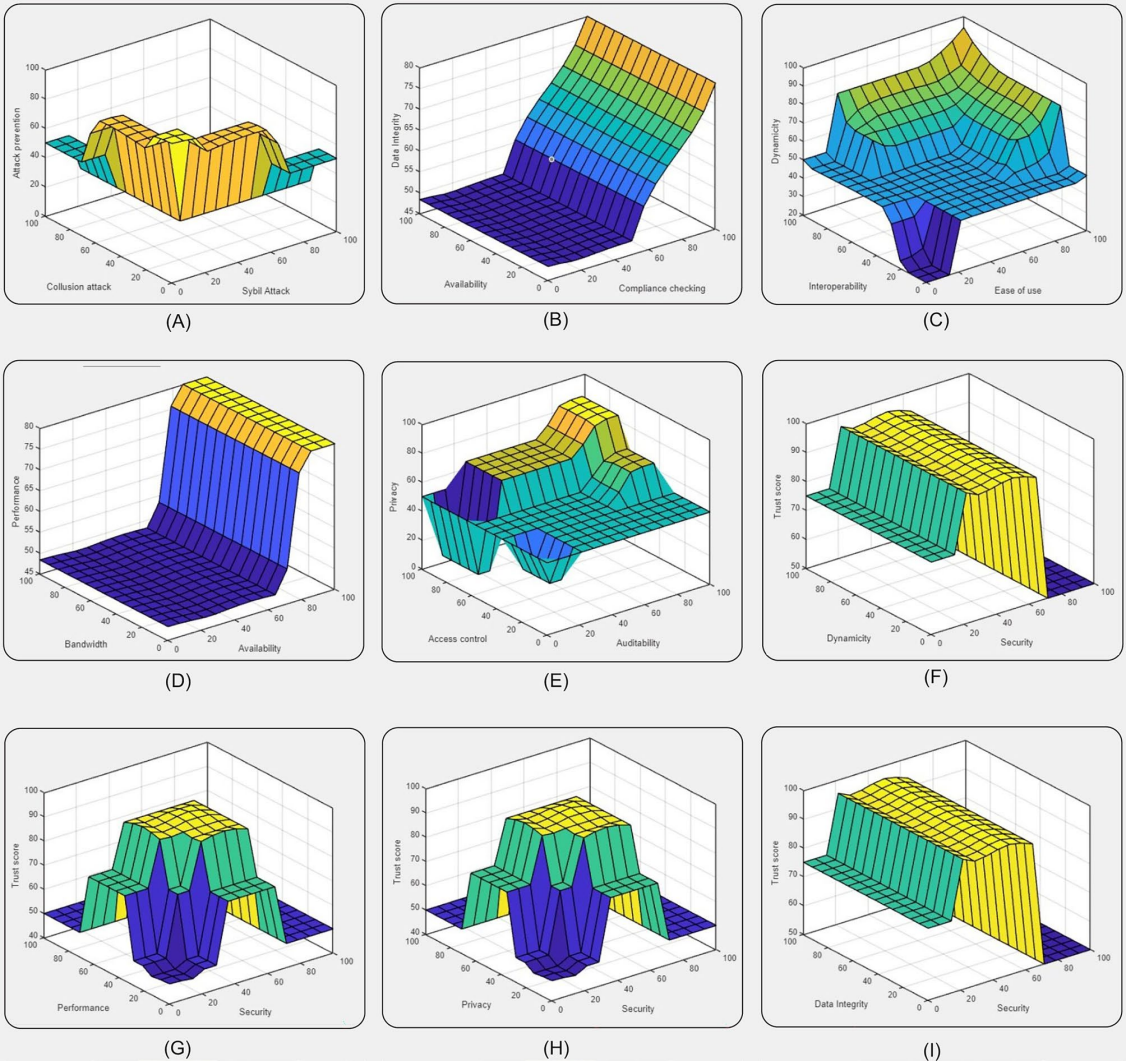
Control Surface of various trust parameters for trust score calculation.

This proposed model is more than well-organized, optimized, and exactitude, where, we custom five parameters such as, security, privacy, dynamicity, data integrity, and performance than other models use different parameters, for industrialized the trust model in cloud computing based on Fuzzy Inference System. Figure 7 shows the surface viewer (SV) of the trust score Fuzzy Inference system. The system’s output surface maps were created and plotted by the surface viewer with the intention of illustrating the link between inputs and outputs. SV is an interactive interface for the FIS which is used to view the output surface of the Fuzzy system depending upon the variation in input values. The generated surface view shows that the increase in the value of major trust parameters may affect the total trust score. In the surface viewer, the output variable increases with an increase in the input variables. That is how it proposes a very optimized way to predict the trustworthiness of a CSP.

### Ranking of the CSP

The CSU can assess each CSP and rank them based on the derived trust score using the fuzzy technique suggested. Assume that the CSU has determined the fuzzy output sets for five distinct CSPs, designated CSP1, CSP2, etc., up to CSP10. The following are the output sets for each CSP: CSP1 = 21.8, CSP2 = 42.9, CSP3 = 29.6, CSP4 = 35.33, CSP5 = 45.5, CSP 6 = 49.56, CSP 7 = 58.52, CSP 8 = 71.5, CSP 9 = 85.1, and CSP 10 = 92.7. The CSP fuzzy output values are shown in the Table 4 according to the input fuzzy sets. Table 4 provides the level 1 parameter values derived from the lower level parameters. The ultimate trust score is calculated using the level 1 factors, and this evaluation determines where the CSP stands among other organizations.

A fuzzy inference system (FIS) that was created to determine a cloud service provider’s trust score by taking important factors like security, privacy, performance, dynamicity, and data integrity into account underwent ablation research. Finding out how each aspect affected the total trust score was the goal Table 5 includes all the characteristics, and the complete model produced a trust score of 0.89. Removing the security parameter resulted in a trust score drop to 0.82, highlighting its significant contribution. Similarly, the exclusion of the performance parameter led to a substantial decrease, bringing the trust score down to 0.80, underscoring its critical importance. Privacy and data integrity, when omitted, caused the trust score to fall to 0.85 and 0.84, respectively, indicating their moderate impact. The dynamicity parameter had a relatively smaller effect, with its removal resulting in a trust score of 0.86. These findings elucidate the varying degrees of influence each parameter has on the trust evaluation, providing valuable insights for refining the FIS to better assess the trustworthiness of cloud service providers.

**Table 5:** Ablation Results for Fuzzy Inference System (FIS).

Removed parameter	Trust score (Full FIS)	Trust score (Ablated FIS)	Change in trust score
None (Full Model)	0.89	–	–
Security	0.89	0.82	− 0.07
Privacy	0.89	0.85	− 0.04
Performance	0.89	0.80	− 0.09
Dynamicity	0.89	0.86	− 0.03
Data Integrity	0.89	0.84	− 0.05

### Rule viewer for trust score evaluation

**Figure 8:**
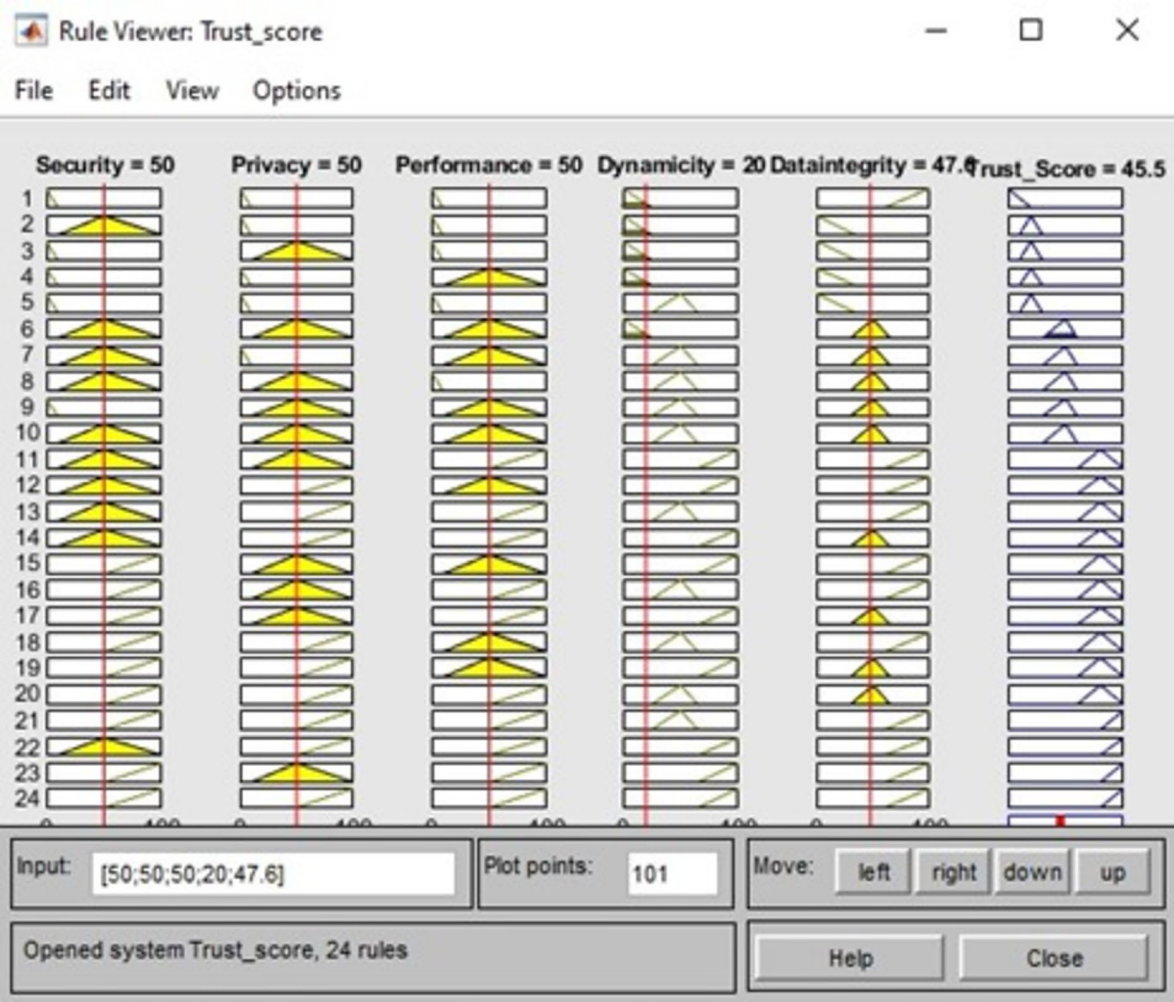
Sample rule viewer for trust score.

For a set of values given to the parameters the final trust score calculated with the relative rules is shown in Fig. 8. To determine the QoS parameters, our proposed model makes use of its provided fuzzy weights. The service’s dependability is then calculated by adding the decision values for each Qos parameter (very high, high, medium, low, and very low). The final trust value rating for a certain service as determined by cloud users is shown in Fig. 7. A triangular is used as a membership function. The membership function shows the relationship between input and output parameters. It is like a mapping in math from input to output. Finally, a comparison of current work with recent works in the same research area was conducted, and the details are included in Table 7.

### Experimental results and comparison of information extraction modules

In order to substantiate our study, we conducted practical experiments to compare various information extraction (IE) modules. These experiments evaluate the performance of each module in extracting relevant trust parameters (security, privacy, performance, dynamicity, and data integrity) from cloud service provider datasets. We evaluated the following IE modules: Module A: Based on rule-based extraction techniques. Module B: Utilizes machine learning algorithms. Module C: Implements a hybrid approach combining rule-based and machine-learning methods. Module D: Employs deep learning models, specifically named entity recognition (NER) and relation extraction.

Table 6 shows the IE module results for various approaches. The rule-based approach achieved good precision but slightly lower recall, indicating it is precise but might miss some relevant information. The machine learning approach improved recall but had moderate precision due to potential overfitting. Also, the hybrid approach balanced precision and recall well, showing significant improvements over pure rule-based or machine-learning methods. In the case of the deep learning approach, it yields the highest F1-score, demonstrating superior performance in accurately extracting relevant trust parameters.

**Table 6:** Experimental Results for Information Extraction Modules.

Module	Precision	Recall	F1-score
A	0.85	0.80	0.82
B	0.88	0.83	0.85
C	0.90	0.87	0.88
D	0.93	0.89	0.91

### Analysis of overhead (time complexity)

The time complexity of the proposed solution is an important aspect to consider, especially in real-time or high-performance computing environments. The overhead introduced by the fuzzy inference system (FIS) primarily depends on several factors, including the number of input parameters, the complexity of membership functions, the size of the rule base, and the computational resources available.

In general, the time complexity of FIS operations, such as fuzzification, rule evaluation, and defuzzification, can be affected by the factors like number of input parameters, complexity of the membership function (triangular membership function), the size of the rule base, and capacity of the computational resources. The time complexity is compared with the machine learning algorithm, in the proposed approach it is comparatively less for an average service provider. It is essential to conduct performance evaluations and benchmarks to assess the actual time complexity to identify potential bottlenecks and optimize the implementation for efficient trust score calculation in cloud computing environments depending upon the various services provided by CSP (Table 7).

**Table 7:** Comparison of Fuzzy-Based Trust Score Calculation with recent works in Cloud Computing.

Aspect	Fuzzy-based trust score calculation	Recent works in cloud computing
Methodology	Utilizes fuzzy inference system to calculate trust scores based on identified parameters	Employs machine learning algorithms for trust prediction in cloud environments
Parameters	Trust parameters identified through literature survey	Trust factors selected based on expert opinion and data analysis
Scalability	Scalable for a large number of trust parameters	Limited scalability due to the complexity of machine learning models
Interpretability	Provides interpretability through linguistic variables and membership functions	Requires additional efforts for interpretability of machine learning models
Customization	Easily customizable by adding or modifying trust parameters	Customization may require retraining of machine learning models
Performance	Offers real-time or near real-time trust evaluation	Performance depends on the complexity of machine learning algorithms

### Performance evaluation (execution time)

To assess the performance of the proposed method based on its execution time, different scenarios with varying workloads can be considered. Case 1: Low complexity evaluation—a small number of Cloud Service Providers (CSPs) with moderate Quality of Service and trust parameters. Case 2: High complexity evaluation- It involves a large number of CSPs with complicated computations and extensive trust parameters. Case 3: Real-time Evaluation- In this case, it uses a real-time trust evaluation for an organization (please refer to case study).

In order to measure the performance of the system different metrics which we can adopt include processing time, fuzzification time, inference time, defuzzification time, etc. The fuzzy logic trust evaluation model is executed in a controlled environment with sample data provided to it. Multiple execution of the model is conducted to capture the variability and compute the various execution times. Table 8 shows sample execution time variations in each scenario.

**Table 8:** Performance evaluation based on execution time.

Scenario	Number of CSPs	QoS parameters	Complexity level	Execution time (s)	Total processing time (s)	Fuzzification time (s)	Inference time (s)	Defuzzification time (s)
Case 1—Low complexity	5	Moderate	Low	10	15	3	6	6
Case 2—High complexity	15	Extensive	High	30	45	8	18	19
Case 3—Real time evaluation	10	Varied, with emphasis on response time	Medium	15	20	5	8	7

### Case study

A company can adopt the trust evaluation model to select CSPs for their critical cloud infrastructure. It can integrate the model into its procurement process, where the potential CSPs can be evaluated based on the model’s trust score derived from the QoS parameter values which can be obtained from their client’s feedback. For each application of their company running on the specific CSP can be evaluated each time based on their trust score. The company can report enhanced decision-making capabilities, as they can now objectively assess and compare CSPs based on trustworthiness. Also by focusing on QoS parameters like security and performance, the model helped mitigate risks associated with selecting unreliable or insecure CSPs.The use of fuzzy logic allowed for transparent representation of uncertain or imprecise information in trust scores, providing a clearer picture to decision-makers.

## Conclusion and future work

In this research, we presented "an optimized trust evaluation model" for cloud trust utilizing a triangular function and a fuzzy mathematics approach. To assess the trust of cloud service providers, we developed the parameter-based trust evaluation method (PBTC). In this research, a novel optimized approach for calculating cloud computing trust is proposed. Using a fuzzy logic system, the trust value of a corresponding source that is cloud-accessible is intended on the source of QoS parameters. Security, privacy, dynamicity, data integrity, and performance make up Quality of Service. For the prospects of future research following improvements can be added into the system. (1) Design a system where more number of parameters and sub-parameters are included. (2) Advanced Fuzzy systems concepts like intuitionistic fuzzy, Neuro fuzzy can be adopted for better results. (3) Machine learning concepts can be adopted to produce real-time values which may produce results that consider feedback also.

To ensure continuous compliance and adaptability, an adaptive framework that integrates ethical considerations into the trust evaluation process can be considered. Real-time Monitoring can be implemented to continuous monitoring of ethical standards and regulations to stay updated with any changes.Various mechanisms can be included to automatically update and adjust the fuzzy inference system (FIS) rules based on new ethical guidelines. Also, we can incorporate context-aware modules that tailor the trust evaluation criteria to specific regulatory and organizational contexts. A feedback loop with stakeholders will help to periodically review and refine ethical standards and compliance measures.

## Data Availability

All the data used is included in the manuscript itself.
